# Fibroblast Growth Factor-2 Induced by Enriched Environment Enhances Angiogenesis and Motor Function in Chronic Hypoxic-Ischemic Brain Injury

**DOI:** 10.1371/journal.pone.0074405

**Published:** 2013-09-30

**Authors:** Jung Hwa Seo, Ji Hea Yu, Hwal Suh, Myung-Sun Kim, Sung-Rae Cho

**Affiliations:** 1 Department and Research Institute of Rehabilitation Medicine, Yonsei University College of Medicine, Seoul, Korea; 2 Graduate Program of Nano Science and Technology, Yonsei University, Seoul, Korea; 3 Brain Korea 21 PLUS Project for Medical Science, Yonsei University, Seoul, Korea; 4 Department of Medical Engineering, Yonsei University College of Medicine, Seoul, Korea; 5 Graduate School of Biomedical Science and Engineering/College of Medicine, Hanyang University, Seoul, Korea; 6 Yonsei Stem Cell Center, Avison Biomedical Research Center, Seoul, Korea; 7 Rehabilitation Institute of Neuromuscular Disease, Yonsei University College of Medicine, Seoul, Korea; Massachusetts General Hospital/Harvard Medical School, United States of America

## Abstract

This study aimed to investigate the effects of enriched environment (EE) on promoting angiogenesis and neurobehavioral function in an animal model of chronic hypoxic-ischemic (HI) brain injury. HI brain damage was induced in seven day-old CD-1® mice by unilateral carotid artery ligation and exposure to hypoxia (8% O2 for 90 min). At six weeks of age, the mice were randomly assigned to either EE or standard cages (SC) for two months. Rotarod, forelimb-use asymmetry, and grip strength tests were performed to evaluate neurobehavioral function. In order to identify angiogenic growth factors regulated by EE, an array-based multiplex ELISA assay was used to measure the expression in frontal cortex, striatum, and cerebellum. Among the growth factors, the expression of fibroblast growth factor-2 (FGF-2) was confirmed using western blotting. Platelet endothelial cell adhesion molecule-1 (PECAM-1) and α-smooth muscle actin (α-SMA) were also evaluated using immunohistochemistry. As a result, mice exposed to EE showed significant improvements in rotarod and ladder walking performances compared to SC controls. The level of FGF-2 was significantly higher in the frontal cortex of EE mice at 8 weeks after treatment in multiplex ELISA and western blot. On the other hand, FGF-2 in the striatum significantly increased at 2 weeks after exposure to EE earlier than in the frontal cortex. Expression of activin A was similarly upregulated as FGF-2 expression pattern. Particularly, all animals treated with FGF-2 neutralizing antibody abolished the beneficial effect of EE on motor performance relative to mice not given anti-FGF-2. Immunohistochemistry showed that densities of α-SMA^+^ and PECAM-1^+^ cells in frontal cortex, striatum, and hippocampus were significantly increased following EE, suggesting the histological findings exhibit a similar pattern to the upregulation of FGF-2 in the brain. In conclusion, EE enhances endogenous angiogenesis and neurobehavioral functions mediated by upregulation of FGF-2 in chronic hypoxic-ischemic brain injury.

## Introduction

Hypoxic-ischemic (HI) brain injury is a major cause of damage to fetal and neonatal brains, and results in considerable morbidity of neurological diseases with neurodevelopmental impairment such as cerebral palsy [1], [2]. HI produces global brain damage in the multiple regions of the hemisphere. Among the regions, the striatum and the cerebellum are main areas involved in maintaining motor coordination and balance. Additionally, the brain areas do not function alone, but particularly interact with the frontal cortex. Because there is a paucity of effective treatments available for adults who have chronic HI brain injury, rehabilitative exercise with exposure to enriched environment (EE) has been a traditional way as a potential treatment to elicit neurorestorative effects in the frontal cortex, striatum, and cerebellum of the brain.

In animal models, EE consisting of running wheels, novel objects, and social interaction has been shown to enhance proliferation of resident neural stem/progenitor cells in the subventricular zone and promote their migration to lesions, contributing to behavioral recovery [3]. Exposure to EE after brain injury has also been shown to provide neuroprotective effects, reducing lesion size and increasing dendritic outgrowth and the production of trophic factors [4]. Exercise is also known to change the morphology of different blood vessels along the arterial tree [5], improving organ blood flow, and causing functional changes [6]. Exercise induces vascular endothelial growth factor (VEGF) [7] and neurotrophins such as nerve growth factor, brain-derived neurotrophic factor (BDNF), and neurotrophin-3 [8], [9]. Especially, fibroblast growth factor-2 (FGF-2), a strong pro-angiogenic factor [10], act as a mediator of the positive effects of exercise on the brain [11]. However, the therapeutic mechanism for how exercise affects the functional outcomes of the brain has been largely unknown.

Therefore, we used an animal model of chronic HI brain injury to investigate 1) whether EE could enhance functional recovery and 2) the therapeutic mechanism by which EE exerts behavioral changes in the multiple regions such as frontal cortex, striatum, and cerebellum. We found that EE elicited neurorestorative effects through the promotion of endogenous repair processes such as angiogenesis and the upregulation of FGF-2 in the fontal cortex and the striatum of the brain.

## Materials and Methods

### Neonatal hypoxic-ischemic brain injury

Permanent ischemic brain damage was induced in 7-day-old CD-1® (ICR) mice by unilateral right carotid artery ligation under anesthesia with ethyl ether. Hypoxic brain injury (8% O_2_ for 90 min) was also generated as previously described [1], [2], [12]. Body temperature was maintained at 37°C while the mice were within the hypoxic chamber (Figure S1A). One week after the HI brain injury, a scalp incision was made in order to locate the brain lesion in the posterolateral area of the right hemisphere.

### Animals and housing

Each experimental group was raised in different conditions. EE mice were housed in a large cage (86×76×31 cm), which contained running wheels, tunnels, shelters, and toys. Standard condition (SC) mice were housed in a standard cage (27×22.5×14 cm) as a control group (Figure S1B, C). At 6 week of age, a total of 30 CD-1® (ICR) mice were randomly assigned to either an EE (n = 15) or standard condition (SC; n = 15) for two months until 14 weeks of age (Figure S1D). Total 30 mice (n = 15 each) which did not receive HI brain injury were also recruited as no HI group to know the EE treatment effect on normal condition and to provide a more comprehensible understanding of the extent of recovery in the subjects [12]. All animals were housed in a facility accredited by the Association for Assessment and Accreditation of Laboratory Animal Care (AAALAC), and given food and water ad libitum with alternating 12-h light/dark cycles.

### Ethics Statement

All procedures were in accordance with the guidelines of the National Institutes of Health's Guide for the Care and Use of Laboratory Animals. These regulations, notifications, and guidelines originated and were modified from the Animal Protection Law (2008), the Laboratory Animal Act (2008), and the Eighth Edition of the Guide for the Care and Use of Laboratory Animals (NRC 2011). The experimental procedure was approved by the Institutional Animal Care and Use Committee (IACUC) of Yonsei University Health System (Permit Number: 2011-0191-1). All animals were maintained in a temperature-controlled animal care facility according to animal protection regulations. They were sacrificed at 2 weeks or 8 weeks after treatment under ketamine (100 mg/kg) and xylazine (10 mg/kg) anesthesia by intraperitoneal injection. Thereafter, they were given an intracardial perfusion of 4% paraformaldehyde, and the brain tissues were harvested. All efforts were made to minimize animal suffering and the number of animals used.

### Behavioral assessment

#### Rotarod performance

A rotarod test was used in order to assess motor coordination and balance. All animals received a pre-operative performance evaluation at five to six weeks of age. Rotarod tests using constant speeds, 48 and rpm were performed at two-week intervals until eight weeks after treatment with EE. The latency of the mice falling from the rod was measured twice during each test, and individual tests were terminated at a maximum latency of 300 sec.

#### Forelimb-use asymmetry test

To evaluate functional asymmetry resulting from a unilateral brain lesion and the resulting hemiplegia, the cylinder test and ladder walking test were performed eight weeks after treatment. In the cylinder test, the number of times each forelimb came into contact with the cylinder wall while the mouse was rearing straight was counted over a period of five minutes. The percentage of hemiplegic forelimb use was evaluated by the following formula [12]:
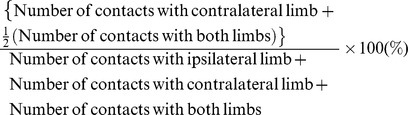



The difference (Δ) in the percentage of the number of cylinder wall contacts by the contralateral limb relative to the preoperative evaluation was calculated.

In the ladder walking test, the mice were required to walk a distance of 1 m three times on a horizontal ladder with metal rungs located different distances apart. The number of slips from the transverse rungs with each forelimb was measured by videotape analysis. The difference (Δ) in the percentage of slips after treatment compared to the preoperative evaluation was calculated by counting the number of slips on the transverse rungs of the ladder relative to the total number of steps taken using the hemiplegic forelimbs.

#### Grip strength test

A grip strength test was performed using the SDI Grip Strength System (San Diego Instruments Inc., San Diego, CA), which includes a push-pull strain gauge. The grip bar was a 2-mm-diameter triangular piece of metal wire. Each animal was held near the base of its tail and brought towards the bar until the animal could grip the bar with its forepaw. The peak force was automatically registered in gram-force by the apparatus. The mean peak force of three trials was used for analysis.

### Immunohistochemistry

Immunohistochemistry was performed as previously described [12]. The brain tissues were frozen and cryosectioned at 16-μm intervals. To confirm endogenous angiogenesis, the brain sections of the frontal cortex and the striatum were immunostained with capillary marker, mouse anti-PECAM-1 (1∶200, Abcam) or smooth muscle actin marker, rabbit anti- α-SMA (1∶200, Abcam). The densities (%) of PECAM-1^+^ and α-SMA^+^ cells in the frontal cortex and the striatum (/mm^2^) were quantified using the MetaMorph Imaging System (Molecular Device, Sunnyvale, CA). PECAM-1^+^ cell density (%) was also measured in the hippocampal region which has already been known to be induced to increase FGF-2 and angiogenesis by physical exercise and EE [11], [13].

### Assessment of growth factors in the brain

To identify the growth factors that are regulated by EE, the frontal cortex, striatum and cerebellum, main regions involved in maintaining motor coordination and balance, were lysed in 200 μl of cold RIPA buffer (50 mM Tris-HCl, pH 7.5, 1% Triton X-100, 150 mM NaCl, 0.1% sodium dodecyl sulfate (SDS), 1% sodium deoxycholate) with a protease inhibitor cocktail (Sigma). Tissue lysates were then centrifuged at 13,000 g for 15 min at 4°C. The supernatant was harvested, and the protein concentration was determined using a protein assay kit (Bio-Rad, Hercules, CA). An array-based multiplex ELISA assay (Mouse Quantibody® array, RayBio-tech, Norcross, GA) was used to determine which of the following 10 mouse cytokines or growth factors were detectable in the frontal cortex: FGF-2, epidermal growth factor (EGF), granulocyte colony-stimulating factor (G-CSF), hepatocyte growth factor (HGF), insulin-like growth factor-1 (IGF-1), leptin (LEP), matrix metalloproteinase-2 (MMP-2), stromal cell-derived factor-1 (SDF-1), vascular cell adhesion protein-1 (VCAM-1), and VEGF. The expression of the angiogenic factors was detected using an array scanner (Gene PIX™ 4000B, Axon instruments, USA).

### Western blot analysis

For electrophoresis, 50 μg of extracted protein from the frontal cortex and the striatum were dissolved in sample buffer (60 mM Tris-HCl, pH 6.8, 14.4 mM β-mercaptoethanol, 25% glycerol, 2% SDS, and 0.1% bromophenol blue), boiled for five minutes, and separated on a 10% sodium dodecyl sulfate (SDS) polyacrylamide gel. Separated proteins were then equally loaded and transferred onto 0.45 μm invitrolon^TM^ polyvinylidene difluoride (PVDF) filter paper sandwich using a XCell II^TM^ Blot Module (invitrogen, Life Technologies, Carlsbad, CA, USA). Blots were blocked for one hour in Tris-buffered saline (TBS) (10 mM Tris-HCl, pH 7.5, 150 mM NaCl) plus 0.05% Tween 20 (TBST) containing 5% non-fat dry milk (Bio-Rad, Hercules, CA, USA) at room temperature, washed three times with TBST, and incubated at 4°C overnight with the following antibodies; anti-FGF-2 (1∶1000, Abcam, Cambridge, UK), anti-activin A (1∶500, Abcam) and anti-GAPDH (1∶1000; Cell Signaling Technology, Beverly, MA, USA) antibodies in TBST (10 mM Tris pH 7.5, 150 mM NaCl, and 0.02% Tween 20) containing 5% non-fat dry milk. The next day, the blots were washed three times with TBST and incubated for one hour with horseradish peroxidase-conjugated secondary antibodies (1∶5000; Santa Cruz, CA, USA) at room temperature. After being washed three times with TBST, the protein was visualized with an enhanced chemiluminescence (ECL) detection system (Amersham Pharmacia Biotech, Little Chalfont, UK).

### RNA preparation

Total RNA was extracted from mouse whole brain and regional (basal ganglia, frontal cortex, hippocampus, and cerebrum) brain using Trizol (Invitrogen Life Technologies, Carlsbad, CA, USA) according to the manufacturers' protocols. The RNA samples were stored at −80°C until further use. For quality control, RNA purity and integrity were evaluated by denaturing gel electrophoresis and OD 260/280 ratio, and analyzed with an NanoDrop Lite spectropotometer (Thermo scientific, Wilmington, DE, USA).

### cDNA synthesis

For cDNA systhesis, total RNA was digested using DNase I (Invitrogen Life Technologies, Carlsbad, CA, USA), synt using the ReverAid first strand cDNA synthesis kit (ThermoFisher scientific, Waltham, MA, USA) according to the manufacturer's instructions. The cRNA was quantified using an NanoDrop Lite spectropotometer (Thermo scientific, Wilmington, DE, USA).

### Quantitative real-time RT-PCR

The qRT-PCR was performed in triplicate on a LightCycler® 480 (Roche Applied Science, Mannheim, Germany) using the LightCycler® 480 SYBR Green master mix (Roche Applied Science, Mannheim, Germany) and the thermocycler conditions were as follows: amplifications were performed starting with a 300-s template preincubation step at 95°C, followed by 45 cycles at 95°C for 10 s, 54°C for 10 s and 72°C for 10 s. The melting curve analysis began at 95°C for 5 s, followed by 1 min at 60°C. The specificity of the produced amplification product was confirmed by the examination of a melting curve analysis and showed a distinct single sharp peak with the expected *Tm* for all samples. A distinct single peak indicates that a single DNA sequence was amplified during qRT-PCR. The primers were as follows: mouse *activin βA*, 5′-ACAGCCAGGAAGACACTGCA-3′ and 5′-CAGGTCACTGCCTTCCTTGG-3′ [14], mouse *GAPDH*, 5′-AACTTTGGCATTGTGGAAG G-3′ and 5′-ACACATTGGGGGTAGGAACA-3′. GAPDH was used as the internal control. The expression of each gene of interest was obtained using the 2^−ΔΔCt^ method.

### Infusion of FGF-2 neutralizing antibody

The mice were infused with 1 μg/ml concentration of FGF-2 neutralizing antibody (Millipore) into the ventricle using Alzet® micro-osmotic pump (model 1002, 0.25 μl/h, 2 weeks; Durect Corporation, Cupertino, CA, USA) so as to inhibit FGF-2 in response to EE or SC. The osmotic pump was changed at 2 week interval until 8 weeks after EE treatment. Mice received HI brain injury at 1 week of age were randomly assigned to either SC/anti-FGF-2 (n = 3) or EE/anti-FGF-2 (n = 4) at 6 weeks of age. Behavioral assessments of these mice were made as described above.

### Statistical analysis

All data were expressed as means ± SEM. To evaluate the effects of EE on the endogenous repair process and functional recovery, statistical analyses were conducted using the Statistical Package for Social Sciences (SPSS) version 20.0. Student's t-test or one-way analysis of variance (ANOVA) was used for the comparison of the variables between EE and SC groups at 2 weeks and 8 weeks following the treatment. A *P*-value <0.05 was considered statistically significant.

## Results

### EE improved rotarod locomotor performance

**Figure 1:**
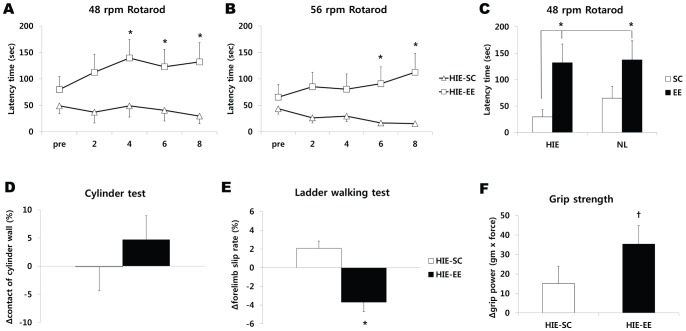
Environmental enrichment improved neurobehavioral function. (A, B) Rotarod tests were performed at 2-week intervals. Rotarod tests at constant speeds (48 rpm and 56 rpm) showed that the rotarod performance of mice treated with EE (HIE-EE) was significantly improved compared to those of HIE-SC until 8 weeks after exposure to EE (^*^ *p*<0.05, n = 15 each). (C) Rotarod performance in mice with no HI brain injury also promoted locomotor function 8 weeks after EE treatment, demonstrating that EE mice with or without brain injury significantly improved 48 rpm rotarod performance relative to the SC mice with HI brain injury (^*^ *p*<0.05, n = 15 each). Other behavioral tests such as the cylinder (D), ladder walking (E) and grip strength tests (F) were performed before EE and at 8 weeks after EE. Ladder walking test showed that percentage of slip late was significantly decreased in the EE mice (^*^ *p*<0.05, ^†^ *p*<0.1, n = 10 each). HIE: hypoxic-ischemic encephalopathy, EE: enriched environment, SC: standard cages.

We first determined whether EE could restore neurobehavioral function using the behavioral rotarod test at two week intervals after the HI brain injury at constant speeds, 48 rpm and 56 rpm. The rotarod results prior to EE showed no differences among the experimental groups. There was a significant improvement in the rotarod performance of the EE mice compared with the SC controls at the constant 48 rpm speed four weeks after treatment (t = 2.287, *p* = 0.032) (Figure 1A) and at the constant 56 rpm speed six weeks after treatment (t = 2.379, *p* = 0.031) (Figure 1B). This significant neurobehavioral improvement was maintained throughout the study period up to post-treatment 8 weeks (t = 2.703, *p* = 0.014 at 48 rpm; t = 2.781, *p* = 0.014 at 56 rpm) (Figure 1A, B).

When we evaluated the rotarod performance in mice with no HI brain injury to know the EE-induced effect on normal condition, they promoted locomotor function 8 weeks after EE treatment in a similar manner (t = 1.717, *p* = 0.099) (Figure 1C). Analysis among four groups demonstrated that EE mice with or without brain injury significantly improved 48 rpm rotarod performance relative to those in SC mice with HI brain injury (one-way ANOVA, F = 3.432, *p* = 0.023). In addition, the EE mice with HI brain injury (132.11±35.29 sec) almost reached to the ability of the undamaged EE mice (137.50±35.96 sec), showing the 96.08% of the locomotor function. On the other hand, the rotarod performance of the SC mice after HI brain injury (29.50±14.0 sec) demonstrated only 45.48% of the locomotor function of the SC mice with no HI brain injury (64.87±22.27 sec).

### EE attenuated forelimb-use asymmetry

To evaluate whether an EE can ameliorate the asymmetry caused by unilateral brain damage, both cylinder and ladder-walking tests were performed at 8 weeks after treatment. In the cylinder test, the percentage of cylinder wall contacts with the hemiplegic forelimb was not statistically different between the EE and SC groups, although the EE mice showed more symmetric pattern of forelimb-use than SC controls (Figure 1C). In the ladder walking test, the percentage of slips on the transverse rungs of the ladder relative to the total number of steps by the hemiplegic forelimbs was significantly decreased in the EE mice (−4.09±0.95%) compared to the SC controls (2.07±0.75%) (t = 5.099, *p*<0.001) (Figure 1D).

### EE tended to improve grip strength

As motor power is characteristically weakened by unilateral ischemic brain damage, we next evaluated whether EE can increase motor strength by measuring grip strength at 8 weeks after treatment. Although there was no statistically significant difference in grip power relative to pre-treatment evaluation between two groups, EE tended to improve the grip strength (35.33±9.37 gram × force) compared to the SC controls (15.10±8.79 gram × force) (t = 1.693, *p* = 0.090) (Figure 1E).

### Effects of EE strongly correlate with upregulation of FGF-2

**Figure 2:**
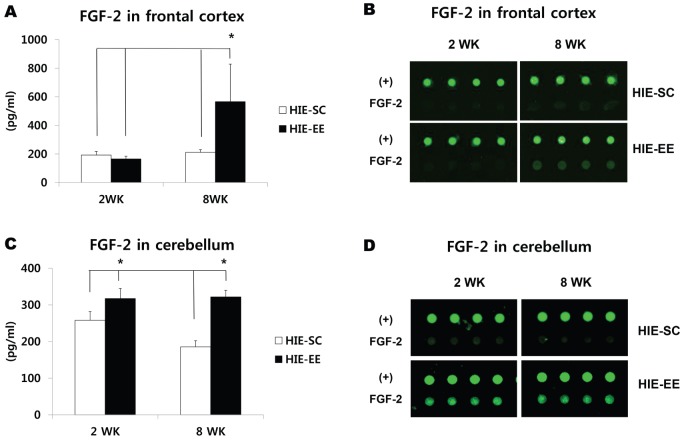
Environmental enrichment strongly upregulated FGF-2 level. (A-D) Two and 8 weeks after exposure to EE, the frontal cortex and cerebellum were lysed, and the levels of various angiogenic growth factors including FGF-2 were determined by multiplex ELISA assay (Quantibody® array). (A) FGF-2 level significantly increased in HIE-EE mice at 8 weeks after treatment (^*^ *p*<0.05, n = 5 each). (B) Representative images of FGF-2 in the frontal cortex using the multiplex ELISA assay. (C) On the other hand, the level of FGF-2 in the cerebellum was upregulated in EE mice at 2 weeks and 8 weeks after treatment (^*^ *p*<0.05, n = 4 each). (D) Representative images of FGF-2 in the cerebellum using the multiplex ELISA assay. HIE: hypoxic-ischemic encephalopathy, EE: enriched environment, SC: standard cages, (+): positive controls.

In order to identify the angiogenic growth factors associated with the repair processes and functional recovery induced by EE, we measured the levels of ten specific candidate factors using an array-based multiplex ELISA assay at both 2 week and 8 weeks after treatment. Among these factors, the level of FGF-2 in the frontal cortex was significantly elevated in mice exposed to an EE (566.0±262.81 pg/ml) compared with SC mice (211.06±19.47 pg/ml) at 8 weeks after treatment (t = 2.178, *p* = 0.043) (n = 5 each). Analysis among the groups demonstrated that only EE for 8 weeks significantly increased the level of FGF-2 in the frontal cortex compared with the other groups (one-way ANOVA, F = 5.362, *p* = 0.004) (Figure 2A). However, other factors such as EGF, G-CSF, HGF, IGF-1, LEP, MMP-2, SDF-1, VCAM-1, and VEGF were not elevated in EE mice compared to the levels in the SC controls (Figure S2). On the other hand, the level of FGF-2 in the cerebellum was upregulated in EE mice compared with SC mice (317.38±14.44 pg/ml versus 258.08±20.75 pg/ml at 2 weeks after treatment, t = 2.346, *p* = 0.057; 321.95±8.0 pg/ml versus 185.49±27.58 pg/ml at 8 weeks after treatment, t = 4.785, *p* = 0.003) (n = 4 each). Analysis among the groups also showed that EE for 2 weeks and 8 weeks significantly increased the level of FGF-2 in the cerebellum (F = 11.222, *p* = 0.001) (Figure 2C).

**Figure 3:**
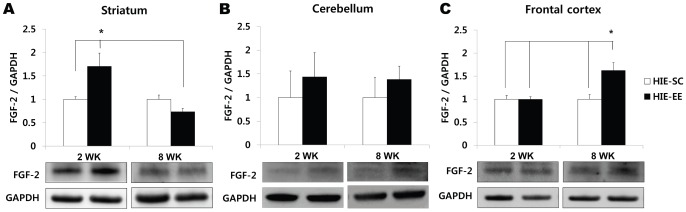
Western blot analysis confirmed FGF-2 upregulation in the brain. (A–C) Analysis of FGF-2 level in the striatum, cerebellum, and frontal cortex using western blotting. (A) FGF-2 significantly increased in the striatum at 2 weeks after exposure to EE (^*^ *p*<0.05, n = 3 each). (B) The FGF-2 in the cerebellum is also highly expressed at 2 weeks and 8 weeks after EE treatment (n = 3 each). (C) On the other hand, the FGF-2 was significantly upregulated in the frontal cortex at 8 weeks after exposure to EE (^*^ *p*<0.05, n = 3 each). HIE: hypoxic-ischemic encephalopathy, EE: enriched environment, SC: standard cages.

Western blotting confirmed this FGF-2 upregulation in the striatum at 2 weeks after exposure to EE (1.70-fold, t = 2.419, *p* = 0.036) (Figure 3A). The FGF-2 in the cerebellum is also highly expressed 1.44-fold at 2 weeks (t = 1.403, *p* = 0.180) and 1.38-fold at 8 weeks after EE treatment (t = 1.832, *p* = 0.089) (Figure 3B). On the other hand, FGF-2 in the frontal cortex significantly increased at 8 weeks after exposure to EE (1.62-fold, t = 3.118, *p* = 0.011). Analysis among the groups also demonstrated that only EE for 8 weeks significantly increased the level of FGF-2 in the frontal cortex compared with the other groups (F = 7.042, *p* = 0.002) (Figure 3C). Taken together, this result suggests that FGF-2 expression in the striatum started to be upregulated earlier than in the frontal cortex. The upregulation of FGF-2 in the frontal cortex, striatum, and cerebellum at 8 weeks post-treatment may have a key role in neurorestorative effect when functional outcomes were maximized in mice with EE.

### EE enhances activin A, a downstream factor for FGF-2

**Figure 4:**
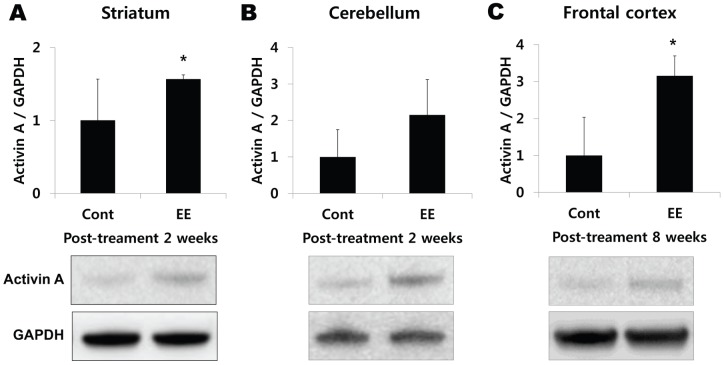
Western blot analysis also confirmed activin A upregulation in the brain. (A–C) Analysis of activin A level in the striatum, cerebellum, and frontal cortex using western blotting. (A) Activin A was significantly upregulated in the striatum at 2 weeks after exposure to EE (^*^ *p*<0.05, n = 3 each). (B) The activin A in the cerebellum is also highly expressed at 2 weeks after EE treatment (n = 3 each). (C) On the other hand, activin A significantly increased in the striatum at 8 weeks after exposure to EE (^*^ *p*<0.05, n = each). HIE: hypoxic-ischemic encephalopathy, EE: enriched environment, SC: standard cages.

Based on a report showed that FGF-2 strongly enhanced the induction of activin A which is essential for the effects of FGF-2 in brain injury [15], we performed real-time PCR and western blotting analysis to check whether expression of activin A was similarly upregulated as a FGF-2 expression pattern. Real-time PCR demonstrated that mRNA of activin A was elevated in cerebral hemisphere at 8 weeks after treatment compared with SC mice (1.72-fold, t = 2.077, *p* = 0.057). When the activin A was also assessed in regional brain using western blotting analysis, it confirmed that activin A was upregulated in striatum (1.74-fold, t = 3.105, *p* = 0.025), cerebellum (2.15-fold, t = 1.643, *p* = 0.131) at 2 weeks after treatment, and in frontal cortex (3.15-fold, t = 3.422, *p* = 0.009) at 8 weeks after exposure to EE (Figure 4A–C). These esults suggest that EE enhances the expression level of activin A in a same pattern as the EE-induced upregulation of FGF-2, which may have a key role in neurorestorative effect by exposure to EE.

### Infusion of FGF-2 neutralizing antibody reverses functional recovery

**Figure 5:**
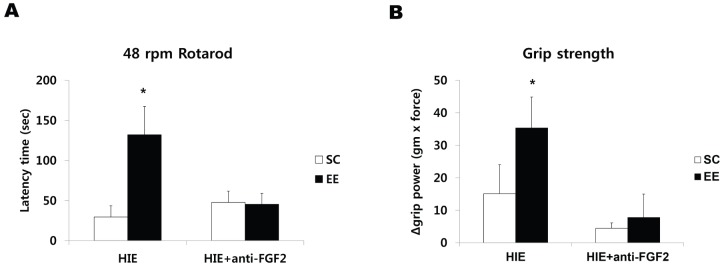
Infusion of FGF-2 neutralizing antibody reversed functional recovery. (A, B) To confirm the relationship between EE-induced FGF-2 upregulation and behavioral benefits, subgroups of EE and SC mice with HI brain injury were randomly assigned to either SC/anti-FGF-2 (n = 3) or EE/anti-FGF-2 (n = 4). (A) All animals treated with anti-FGF-2 abolished the beneficial effect on motor performance relative to EE mice not given anti-FGF-2 at 8 weeks after treatment (^*^ *p*<0.05). (B). Mice treated with EE/anti-FGF-2 also showed the decreased grip strength whereas grip strength of EE mice not given anti-FGF-2 was significantly increased compared to the other groups (^*^ *p*<0.05).

To confirm the relationship between EE-induced FGF-2 upregulation and behavioral benefits, we assessed the effects of EE while simultaneously inhibiting FGF-2. Subgroups of EE and SC mice with HI brain injury were infused chronically with a FGF-2 neutralizing antibody. We demonstrated that all animals treated with anti-FGF-2 abolished the beneficial effect of EE on motor performance when evaluated with mice not given anti-FGF-2. Namely, mice treated with EE/anti-FGF-2 exhibited the same rotarod latency (47.6±10.8 sec) as those of SC (29.50±14.0 sec) and SC/anti-FGF-2 (52.4±11.9 sec) whereas rotorod latency in EE mice not given anti-FGF-2 (132.11±35.29 sec) was significantly increased compared to the other groups 8 weeks after treatment (one-way ANOVA, F = 4.345, *p* = 0.009) (Figure 5A). Additionally, mice treated with EE/anti-FGF-2 showed the decreased grip strength (7.78±3.76 gram × force) as those of SC (15.1±8.79 gram × force) and SC/anti-FGF-2 (4.44±3.62 gram × force) whereas grip strength of EE mice not given anti-FGF-2 (35.33±9.37 gram × force) was significantly increased compared to the other groups 8 weeks after treatment (F = 2.917, *p* = 0.038) (Figure 5B).

### EE enhances endogenous angiogenesis

**Figure 6:**
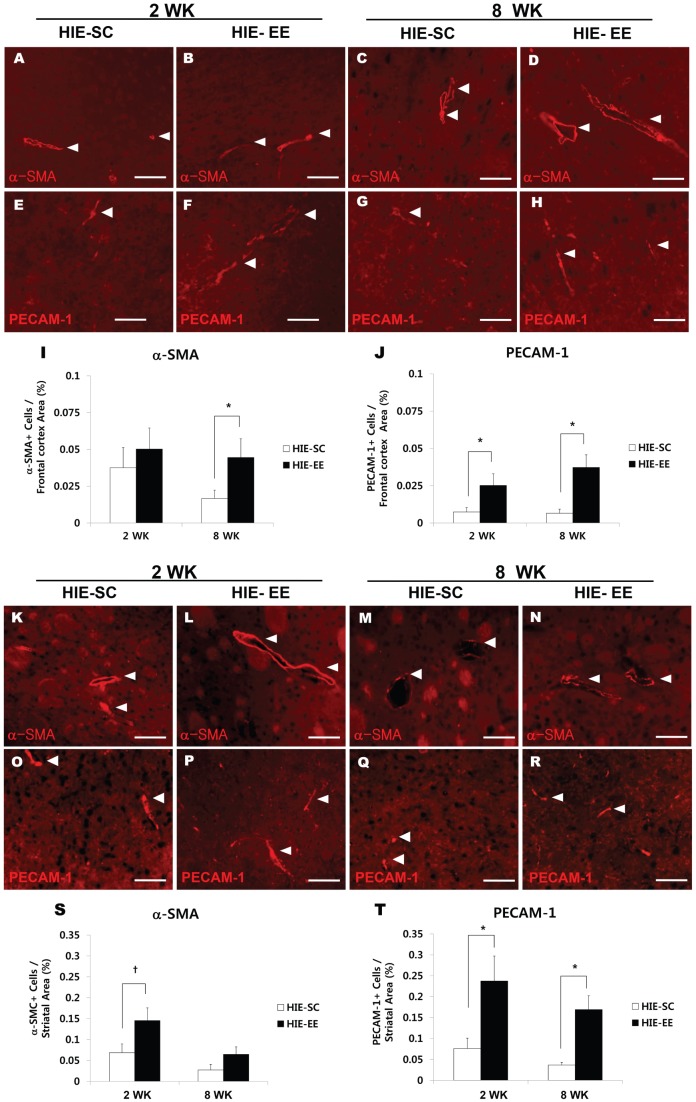
Environmental enrichment enhanced endogenous angiogenesis in the frontal cortex and striatum. (A–H, K–R) Two and 8 weeks after exposure to EE, the densities of α-SMA^+^ cells and PECAM-1^+^ cells were quantified using the MetaMorph Imaging System. Scale bar 50 μm. (I) The density of α-SMA^+^ cells was significantly higher in frontal cortex of EE mice than SC controls 8 weeks after treatment (^*^ *p*<0.05, n = 5 each). (J) The HIE-EE mice also showed an increase in PECAM-1^+^ angiogenesis 2 weeks and 8 weeks after treatment (^*^ *p*<0.05, n = 5 each). (S, T) The densities of α-SMA^+^ cells (S) and PECAM-1^+^ cells (T) were higher in the striatum of EE mice than SC controls at 2 weeks after treatment (^*^ *p*<0.05, ^†^ *p*<0.1, n = 5 each). (T) The densities of PECAM-1^+^ cells were also significantly increased at 8 weeks following the EE treatment (^*^ *p*<0.05, n = 5 each). HIE: hypoxic-ischemic encephalopathy, EE: enriched environment, SC: standard cages.

**Figure 7:**
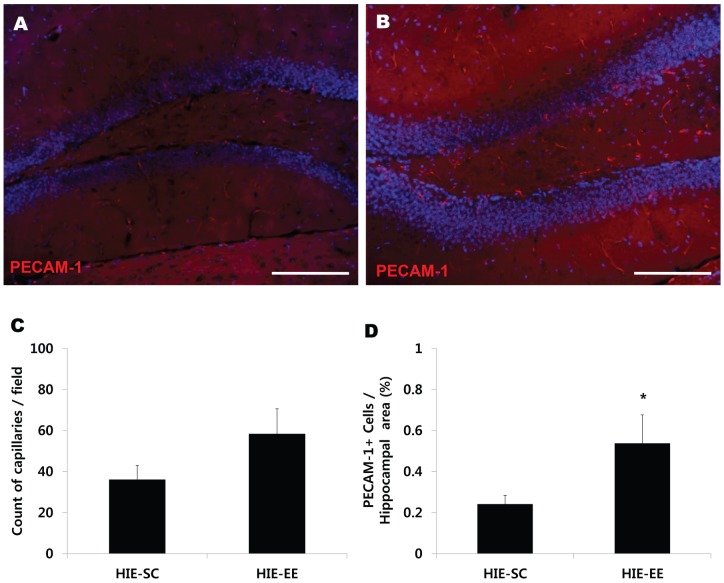
Environmental enrichment also enhanced endogenous angiogenesis in the hippocampus. (A, B) After exposure to EE, the capillary count and densities of PECAM-1^+^ cells were quantified using the MetaMorph Imaging System. Scale bar 150 μm. (A) Capillary count was increased in the dentate gyrus of the hippocampus at 8 weeks following the exposure to EE. (B) In particular, the densities of PECAM-1 in the EE mice were significantly higher than those in the SC controls at 8 weeks after treatment (^*^ *p*<0.05, n = 5 each). HIE: hypoxic-ischemic encephalopathy, EE: enriched environment, SC: standard cages.

The effect of treatment with EE in inducing endogenous angiogenesis in the frontal cortex, striatum, and hippocampus was assessed immunohistologically by quantifying the densities of α-SMA^+^ and PECAM-1^+^ cells at both 2 week and 8 weeks after treatment (n = 5 each) (Figure 6 and Figure 7). Two weeks after treatment, the group treated with EE started to show an increase in PECAM-1^+^ angiogenesis in the frontal cortex (0.025±0.007%, t = 2.238, *p* = 0.046) compared with the SC controls (0.007±0.003%) (Figure 6J). Eight weeks post-treatment, the densities of α-SMA (0.045±0.012%, t = 2.105, *p* = 0.050) and PECAM-1 (0.037±0.008%, t = 3.619, *p* = 0.004) in the frontal cortex were significantly increased in the EE mice compared with the SC controls (0.017±0.005%, 0.007±0.002% respectively) (Figure 6I, J). Analysis among the groups also showed that EE for 2 weeks and 8 weeks significantly increased the densities of PECAM-1 in the frontal cortex (one-way ANOVA, F = 6.522, *p* = 0.001) (Figure 6J).

In the striatum, the densities of α-SMA (0.145±0.030%, t = 2.083, *p* = 0.052) and PECAM-1 (0.238 ± 0.060%, t = 2.495, *p* = 0.023) were also increased in the EE mice compared with the SC controls (0.069±0.021%, 0.076±0.025% respectively) at 2 weeks after treatment (Figure 6S, T). Likewise, the densities of PECAM-1 (0.169±0.033%) in the striatum were significantly increased in the EE mice at 8 weeks after treatment, compared with the SC controls (0.037±0.007%) (t = 3.920, *p* = 0.003) (Figure 6T). Analysis among the groups demonstrated that EE for 2 weeks significantly increased the striatal densities of α-SMA (F = 5.257, *p* = 0.004) and PECAM-1 (F = 6.186, *p* = 0.002) (Figure 6S, T). Additionally, the capillary count was increased in the dentate gyrus of the hippocampus at 8 weeks following the exposure to EE (Figure 7A). In particular, the densities of PECAM-1 (0.5363±0.133%) in the EE mice were significantly higher than those in the SC controls (0.240±0.041%) (t = 2.132, *p* = 0.047) (Figure 7B). Taken together, the histological findings of this study exhibit a similar pattern to the upregulation of FGF-2 in the brain.

## Discussion

Previous studies have demonstrated the beneficial effects of exercise in improving balance, muscle strength, ambulatory function, and cardiovascular fitness in patients with stroke and Parkinson's disease [16], [17]. In addition with effects on physical outcomes, EE significantly improves brain plasticity and functional outcomes with expression of neurotrophic factors and neurogenesis in animal models of ischemic stroke [3], [4], [18], Huntington's disease [19], and Alzheimer's disease [20]. Several studies have reported that EE can enhance learning and memory, neurogenesis in the dentate gyrus [21], [22], gliogenesis [23], neurite branching [23], [24], and increase the levels of growth factors and neurotransmitters in the cortex and hippocampus [25], [26]. Exercise also upregulates neurotrophic factors including BDNF and IGF-1, which may render brain tissue resistant to degenerative events [27]. Animals that exercise after brain injury show an increase in the expression of neurotrophic factors, such as BDNF, HGF, and FGF-2, which regulate neuronal survival and differentiation, synaptic plasticity, as well as angiogenesis in the brain [28], [29].

In this study, we examined the restorative effects of EE consisting of running wheels, novel objects, and social interaction at five weeks after neonatal HI brain injury, the equivalent of six-week adult age. The main purpose of our study was to verify the therapeutic mechanism by which EE induces functional recovery in an animal model of chronic HI brain injury. We demonstrated that, eight weeks after treatment with EE, neurorestorative effects and maximal functional outcomes were seen in various behavioral assessments such as rotarod performance and ladder walking tests. The grip strength test also revealed that EE could systemically improve bilateral grip strength. In particular, the expression of FGF-2 among various angiogenic growth factors was enhanced in the frontal cortex of EE mice at post-treatment eight weeks when significant functional recovery was disclosed.

Multiplex ELISA demonstrated that only FGF-2, but not other factors, was significantly elevated in mice exposed to EE compared with SC mice. Therefore, the upregulation of FGF-2 might be an important factor in functional recovery.

Housing animals in an EE several weeks before brain injury results in a 50% increase in the expression of FGF-2 and attenuates functional deficits [30]. Moreover, forced use of one forelimb by constraining the other forelimb upregulates the expression of FGF-2 [31]. FGF-2 is also a potent chemotactic factor for endothelial cells [32], and it plays a role in modulating recovery from cerebral injury [33], [34]. FGF-2 was found to improve sensorimotor deficits and to reduce infarct size following cerebral ischemia in adult rats [35], and neutralizing antibodies to FGF-2 blocks recovery from motor cortex lesions [36]. The role of FGF-2, which was significantly upregulated in the present study, as a mediator of the effects of exercise on the brain is supported by demonstrations that FGF-2 is not only a strong pro-angiogenic factor [10], but is also a neurotrophic factor in the adult brain [34], [37]. FGF-2 can be induced by physical exercise and regulated in an activity-dependent fashion, which increases the possibility that FGF-2 is involved in behavioral function [11].

As a therapeutic mechanism, exercise induces angiogenesis in motor cortex of the rat [38], [39]. Treadmill exercise has been demonstrated that it could induce striatal angiogenesis and reduce neurologic deficits in ischemic rats [40]. The exercise-induced angiogenesis is specific to areas activated by the training [39]. Voluntary physical activity also improves long-term stroke outcome related with augmentation of angiogenesis and cerebral blood flow within the ischemic striatum [41]. In patients with ischemic stroke, the higher cerebral blood vessel counts correlated with longer survival [42]. Collateral growth and new capillaries support restored perfusion in the ischemic border after stroke and promote functional recovery [43].

The present study showed that the densities of PECAM-1^+^ and α-SMA^+^ cells in the frontal cortex and the striatum of EE mice were significantly increased after treatment, demonstrating a similar pattern to the upregulation of FGF-2. However, the newly generated vessels were not shown in the frontal cortex and the striatum, when stained with PECAM-1 and α-SMA, and bromodeoxyuridine (BrdU) in an additional group of subjects received an i.p. injection of BrdU (50 mg/kg) once a day for 12 days beginning one day after exposure to an EE (Figure S3). This result suggests that endogenous angiogenesis might be mediated by capillary sprouting, bridging, and intussusception from pre-existing vessels rather than the newly generated vessel formation after the EE treatment. Because vascular smooth muscle cells play critical roles in vascular maturation and arteriogenesis while endothelial cells can initiate angiogenesis [44], [45], overexpression of both endothelial cell marker PECAM-1 and smooth muscle cell marker α-SMA shown in this study suggests that EE enhances the process from angiogenic initiation to vascular maturation.

Taken together, EE enhances endogenous angiogenesis and neurobehavioral functions mediated by the mechanism of upregulation of FGF-2. Interestingly, FGF-2 in the striatum significantly increased at 2 weeks after exposure to EE earlier than the increment at 8 weeks after treatment in the frontal cortex. The striatum is strongly connected with the cerebral cortex, and are associated with a variety of functions, including posture and motor control, coordination, locomotion, procedural learning, and cognitive and emotional functions [46], [47]. This study suggests that the striatum is sensitively affected by EE which contains a complex of physical, cognitive, and social stimuli. The area in the brain may play an important role in functional recovery by rehabilitative exercise [48].

## Conclusion

The upregulation of FGF-2 induced by EE promoted functional recovery through enhanced angiogenesis in an animal model of chronic HI brain injury. Our data confirmed the established link between neurobehavioral/histological outcomes and the upregulation of FGF-2 following EE. Taken together, our results suggest that a rehabilitative strategy with EE could be effective for the treatment of CP, and that it may also be applied to the treatment of other neurological diseases including adult ischemic stroke.

## Supporting Information

Figure S1 Experimental design. (A) After ischemic brain damage was induced by unilateral right carotid artery ligation in 7-day-old CD-1® (ICR) mice, hypoxia was induced at 8% O2 for 90 min using nitrogen gas (N2) and monitored using oximetry. After, at postnatal week 6 (P42), mice were housed in (B) standard cage (27×22.5×14 cm) or (C) enriched environment (86×76×31 cm) including tunnels, shelters, toys, running wheels for voluntary exercise, and social interaction. (D) Schematic timeline of the experimental procedures.
(EPS)

Figure S2 **Angiogenic growth factor expression in the frontal cortex.** (A–I) Two and 8 weeks after exposure to EE, the frontal cortex was lysed, and the levels of various angiogenic growth factors were determined by multiplex ELISA assay (Quantibody® array). However, EE did not upregulated various growth factors except the FGF-2. HIE: hypoxic-ischemic encephalopathy, EE: enriched environment, SC: standard cages.
(EPS)

Figure S3 **Environmental enrichment did not generate new vessels.** (A, B) When stained with PECAM-1 and α-SMA, and bromodeoxyuridine (BrdU) in an additional group of subjects received an i.p. injection of BrdU (50 mg/kg) once a day for 12 days beginning one day after exposure to an EE, the newly generated vessels were not shown in the frontal cortex and the striatum, suggesting that endogenous angiogenesis might be mediated by capillary sprouting, bridging, and intussusception rather than the newly generated vessel formation after the treatment.
(EPS)
